# Supplementary material for: MetaRibo-Seq measures translation in microbiomes
Source: Nat Commun. 2020 Jun 29;11:3268. doi: 10.1038/s41467-020-17081-z (PMC7324362; doi:10.1038/s41467-020-17081-z)
Supplement: Supplementary file 10 — Supplementary Data 7 [file 41467_2020_17081_MOESM10_ESM.zip › File2/Confidence_High_Taxonomy/224637_out.krona.html]

Javascript must be enabled to view this page.

members
magnitude
magnitudeUnassigned
count
unassigned
taxon
rank

224637\_out

2

superkingdom
2
2

2
1239
phylum

class
2
186801

order
186802
2

family
541000
2

2
216851
genus


SRS015663\_contig\_number\_22938SRS052697\_contig\_number\_8155
species
2
853
